# The Impact of Electron Phonon Scattering, Finite Size and Lateral Electric Field on Transport Properties of Topological Insulators: A First Principles Quantum Transport Study

**DOI:** 10.3390/ma16041603

**Published:** 2023-02-15

**Authors:** Elaheh Akhoundi, Michel Houssa, Aryan Afzalian

**Affiliations:** 1 IMEC, B-3001 Leuven, Belgium; 2 Department of Physics, KU Leuven, B-3001 Leuven, Belgium

**Keywords:** atomistic modeling, topological insulators, quantum transport, low-dimensional material

## Abstract

We study, using non-equilibrium Green’s function simulations combined with first-principles density functional theory, the edge-state transport in two-dimensional topological insulators. We explore the impact of electron–phonon coupling on carrier transport through the protected states of two widely known topological insulators with different bulk gaps, namely stanene and bismuthene. We observe that the transport in a topological insulator with a small bulk gap (such as stanene) can be heavily affected by electron–phonon scattering, as the bulk states broaden into the bulk gap. In bismuthene with a larger bulk gap, however, a significantly higher immunity to electron–phonon scattering is observed. To mitigate the negative effects of a small bulk gap, finite-size effects are studied in stanene ribbons. The bulk gap increases in ultra-narrow stanene ribbons, but the transport results revealed no improvement in the dissipative case, as the states in the enlarged bulk gaps aren’t sufficiently localized. To investigate an application, we also used topological insulator ribbons as a material for field-effect transistors with side gates imposing a lateral electric field. Our results demonstrate that the lateral electric field could offer another avenue to manipulate the edge states and even open a gap in stanene ribbons, leading to an *I*_ON_/*I*_OFF_ of 28 in the ballistic case. These results shed light on the opportunities and challenges in the design of topological insulator field-effect transistors.

## 1. Introduction

Topological insulators (TIs) are a class of materials with unique transport properties. Two-dimensional (2D) TIs possess helical edge states that are protected against elastic back-scattering [1,2]. The prohibited backscattering is linked to the bulk electronic structure and the time-reversal symmetry. Even with imperfections (such as defects), the protection of edge states is still preserved [3,4].

As an important step, the quantized conductance of the helical states should be observed experimentally. This has been challenging and the success has been limited to very low temperatures or short protection lengths [5,6]. This has been linked to different backscattering mechanisms, such as edge disorder [7], unprotected low-energy edge states [8], spontaneous time-reversal symmetry breaking [9], charge puddles that trap edge carries [10], charge dopants [11], and phonon scattering [12]. In particular, the effects of electron–phonon scattering on the transport properties of TIs have been neglected in most previous work.

The quantum confinement effects on the electronic properties of a few 2D TIs have been studied [13,14,15]. Additionally, the finite size effects on the gate-induced phase transition in 2D Xenes have been explored using Kane-Mele Hamiltonian [15]. Quantum confinement can result in an increase in the bulk gap, as well as a gap opening in the metallic edge states due to the hybridization between the states corresponding to the two opposite edges of the ribbon [16]. However, the crossing point and anti-crossing point in zigzag Xene ribbons are located at different momentum space locations, and the edge states remain gapless in these ribbons despite a finite inter-edge overlapping [15]. Additionally, a reduction in the critical electric field of the ultra-narrow zigzag Xenes is expected [15]. To further understand these features, the transport properties of ultra-narrow Xene ribbons need to be explored.

The application of 2D TIs in field-effect transistors (TI-FETs) has been investigated extensively, and a few switching mechanisms have been put forward. TI-FETs based on scattering modulation have been proposed and studied using the Kane-Mele tight-binding (TB) Hamiltonian [3,17]. In this case, the shift in the Fermi level from the protected states to the unprotected states leads to the intensification of the scattering effects, resulting in a smaller current. An on/off current ratio of two orders of magnitude has been reported for an imperfect stanene ribbon with an optimized defect density in the ballistic regime by using TB and non-equilibrium Green’s function (NEGF) formalism [3].

Additionally, a gate-induced electric field can cause a topological phase transition and open a gap in the metallic edge states. The critical electric field for such a transition has been reported for several TIs theoretically and experimentally. For instance, critical fields of 1 V/nm for stanene [18], 1.42 V/nm for 1T’-MoS_2_ [19], and 1.1 V/nm for Na_3_Bi [20] have been reported, all of which are undesirably large. In addition, although a phase transition occurs at these fields in the monolayer, a sufficient gap may only open at an even larger electric field in the nanoribbon (NR).

In previous work, atomistic simulations were used to study TI-FETs based on an out-of-plane electric-field-induced phase transition in 1.28 nm wide Na_3_Bi ribbons [16]. The authors reported an ON/OFF current ratio of 6/4 at *V*_bias_ of 0.05 V/0.1 V, respectively. The simulations were performed in the ballistic case and with a channel length of 2.7 nm. Additionally, a differential voltage *V*_diff_ (between the top and bottom electrodes, *V*_top_ and *V*_bottom_, respectively), of around 20 V was required to switch off the TI-FET. In another work based on Na_3_Bi ribbons, the use of an electric field is coupled with the intrinsic defects for improving the device performance by disorder filtering [21].

Moreover, a transverse electric field can offer another approach to designing a transverse tunneling transistor, by taking advantage of the inter-edge tunneling modulated by such a field [22]. In addition, a lateral electric field applied across the edges can be utilized to manipulate the edge channels [23]. Compared to other switching mechanisms, less attention has been given to the application of side gates in TI devices.

Here, we used density functional theory (DFT) based NEGF, implemented in our ATOmistic MOdeling Solver (ATOMOS) [24,25], to explore the influence of electron–phonon (e-ph) scattering on the edge state transport in TIs. The simulations include wide ribbons, heavy elements, and spin–orbit coupling. Therefore, we used a Hamiltonian size reduction method known as mode space (MS) transformation to reduce the computational burden of the calculations [26,27,28]. Using ATOMOS, we investigated the finite size effects on the transport properties of zigzag stanene ribbons as a small bulk gap TI and explored the concept of a field-effect transistor operating based on a lateral electric field introduced by side gates.

In Section 2, we summarize the computational methods used in our simulations. We present and discuss the results in Section 3 and conclude in Section 4.

## 2. Materials and Methods

First principles calculations based on DFT were carried out using the OpenMX simulation tool [29,30]. Generalized gradient approximation (GGA) and sets of pseudo-atomic orbital basis functions of Bi8.0-s3p2d2 and Sn11.0-s3p2d2 were used to perform band structure calculations and geometry relaxation. Spin–orbit coupling was included. The K-point mesh was 12 × 12 × 1 for the edgeless (periodically infinite) monolayers and 14 × 1 × 1 for the edged nanoribbons. A cut-off energy of 200 Ry was used for charge density. A uniform external electric field in a sawtooth waveform was applied. A vacuum of approximately 15 Å was considered to cut off the periodic image in the non-periodic directions. The momentum (*k*)-resolved spin splitting was obtained using the post-processing code “kSpin” [30]. All the structures were fully relaxed with an atomic force convergence criterion of 10^−3^ eV/Å.

The Hamiltonian and overlap matrix elements obtained from the DFT simulations were used to build the full device Hamiltonian. DFT—based NEGF calculations were carried out self-consistently using ATOMOS. The complete simulation procedure is detailed in [25,31]. An MS approach was used to fasten the simulations [27]. The scattering self-energies were not computed in mode space using a form factor method, but directly in real space using the up-converted lesser and greater Green’s functions, followed by a down-conversion of the scattering self-energies to mode space [32,33].

We used the self-consistent Born approximation, based on DFT-calculated deformation potential constants and assuming equilibrium phonons [32,33], to study the effects of electron–phonon scattering on carrier transport in TIs. It has been reported that one needs to only consider acoustic phonon scattering in stanene, as the scattering rate of (inelastic) optical phonons is significantly smaller [34]. In addition to the acoustic elastic phonons, we also considered here, for completeness, a weak optical phonon scattering mechanism with constant parameters in all the simulations (except for the ballistic transport simulations). The optical deformation constant was set to 4 eV/nm with a constant phonon energy of 49 meV.

The acoustic deformation potential constant for a free-standing monolayer stanene with iodine as surface functionalization is reported to be 27 eV for transverse and longitudinal acoustic phonons [34]. In this work, our focus is to quantify the presence or absence of protection against backscattering depending on the TI material properties (e.g., the bulk gap) and investigate how it is affected by the scattering parameters. For that purpose, we varied the acoustic deformation potential from 0 to 80 eV, i.e., significantly smaller and larger than the calculated free-standing value (27 eV). The quantification of how much the scattering parameters of the materials considered here would be impacted by the gate oxide or other interfaces or defects was not considered here and is out of the scope of this paper.

To study the effects of electron–phonon scattering, confinement, and electric field, we focused on two widely studied Xene TIs, stanene (with a bulk gap of 0.17 eV [17]) and bismuthene (with a bulk gap of 0.5 eV [4]).

## 3. Results

### 3.1. Zigzag Nanoribbons of Stanene and Bismuthene

Here, we compare the influence of electron–phonon scattering on the transport properties of two Xenes (stanene and bismuthene), which are well-known topological insulators with different bulk gaps. To study the edge state transport, the zigzag nanoribbons of these two materials are considered.

#### 3.1.1. Mode Space Basis

**Figure 1:**
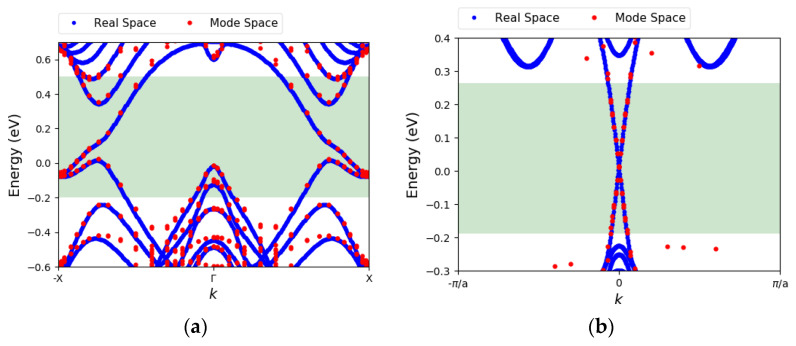
(**a**) The electronic band dispersion of 4 nm wide zigzag stanene ribbon with fluorine edge passivation, calculated by real space (RS) Hamiltonian and mode space (MS) Hamiltonian. The green-shaded region indicates the target energy window (**b**) same as (**a**) for a 7 nm wide zigzag bismuthene ribbon with H edge passivation.

A mode space (MS) method is employed to build the device Hamiltonian. Using this method, the Hamiltonian size is considerably reduced, and the band structure is reproduced accurately in an energy window of interest. Figure 1a illustrates the mode space band structure obtained for a 4 nm wide zigzag stanene ribbon. The blue and red dots represent the bands computed with the real space (RS) and mode space Hamiltonians, respectively. The shaded green region represents the energy window of interest. This energy window contains the protected edge states, as well as a few unprotected bulk bands. This energy window is sufficient for our simulations as the drain bias voltage (*V*_DD_) used for stanene will be small to ensure the transport inside the small bulk gap. The helical spin texture of the protected bands imposes a spin-momentum locking which impedes the elastic backscattering in the ribbon [35]. The bulk band gap appears to be 0.34 eV for this 4 nm wide structure. It is larger than the monolayer bulk band gap due to the confinement effects that we will further discuss in the next section.

Figure 1b displays the band structure for a 7 nm wide zigzag bismuthene ribbon obtained from the RS and MS Hamiltonians. The bulk band gap is ~0.55 eV. An amount of 7 nm is wide enough that the bulk gap value approaches that of the monolayer. As shown in Figure 1b, the selected energy window only covers the edge states. That is because the bulk gap is larger in bismuthene and by tuning both the drain bias and the Fermi level, we can expect that the transport will only take place through the edge states.

#### 3.1.2. The Effects of Electron–Phonon Coupling

**Figure 2:**
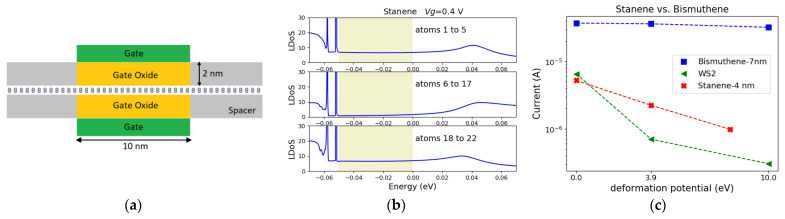
(**a**) An illustration of the structure under study. (**b**) The atom resolved LDoS for the structure based on 4 nm wide stanene zigzag ribbon in the ballistic regime. The contribution of the left edge atoms (atoms 1 to 5), middle atoms (atoms 6 to 17), and right edge atoms (atoms 18 to 22) to the LDoS are demonstrated. The shaded region denotes the Fermi window. (**c**) The calculated current for the structures is based on the stanene and bismuthene zigzag ribbons as well as a WS_2_ monolayer (in H-phase). The current is plotted as a function of *D*_AC_.

To study the effects of e-ph coupling, we considered the TI-FET device depicted in Figure 2a. The structure consists of a TI ribbon (stanene or bismuthene) subjected to a top and a bottom gate. The gates modulate the Fermi level. If the Fermi level lies inside the bulk gap, the carriers transport through the protected edge states. Once the Fermi level is outside the bulk gap, the bulk carriers participate in the current, resulting in a dissipative transport. The channel length (*L_ch_*) is 10 nm for all the structures in this work. For stanene NR, the drain bias is 50 mV. The drain bias is chosen to be small due to the small bulk gap of stanene.

After constructing the device Hamiltonian and performing the self-consistent NEGF simulations with ATOMOS, the local density of state (LDoS) is obtained. LDoS is extracted from the middle of the device. The bands are shifted downward as the gate voltage, *V*_g_, increases. At *V*_g_ = 0.4 V, the entire Fermi window (the shaded area in Figure 2b) is covered by the protected edge states. Figure 2b illustrates the contribution of the left edge atoms (atoms 1 to 5), the middle atoms (atoms 6 to 17), and the right edge atoms (atoms 18 to 22) to the LDOS at *V*_g_ = 0.4 V. It can be observed that the low-energy states inside the bulk gap are localized at the edges of the ribbon, while the states outside the gap, as well as high-energy sates inside the bulk gap, are delocalized.

The inclusion of e-ph scattering in the simulation results in a typical broadening of the bulk states into the bulk gap and consequently into the Fermi window [35]. Here, the acoustic phonon scattering is taken into account and different effective deformation potential constants (*D*_AC_) are compared to represent different e-ph coupling strengths. As *D*_AC_ increase, the Fermi window gets fully covered with the broadened, unprotected states at *D*_AC_ = 6 eV [35]. Therefore, the backscattering is not prevented as the protected states can’t be isolated. This results in a noticeable current degradation.

On the other hand, bismuthene has a larger bulk gap. Simulations with the same structure and gate setup as in Figure 2a were performed on a 7 nm wide bismuthene zigzag nanoribbon. A larger source-drain bias of 0.2 V was considered for bismuthene. The gate work functions were adjusted to achieve the same conduction band shift vs. flat band at *V*_g_ = 0 V for both channel materials. At *V*_g_ = 0.4 V, in both cases, the Fermi window is covered by the protected edges. As bismuthene possess a larger bulk gap, the edge localization of the protected states is preserved in bismuthene even for a *D*_AC_ as large as 40 eV [35]. Hence, it is expected that bismuthene exhibits better immunity to e-ph coupling. The calculated currents for the structures based on stanene and bismuthene as well as WS_2_ monolayer (in H-phase) are shown in Figure 2c. As the deformation potential increases from 0 to 10 eV, an important current degradation is observed for the stanene and WS_2_ monolayers, while the transport in bismuthene is almost unaffected.

Since the broadening of the bulk states and the small bulk gap in stanene are the main causes of the current degradation (and not the edge-to-edge tunneling), increasing the width of the ribbon will not solve this problem. On the contrary, a width reduction could result in a bulk gap increase due to quantum confinement. This, in turn, may enhance the stanene ribbon transport immunity to e-ph scattering and will be investigated in the next section.

### 3.2. Ultra-Narrow Ribbons

**Figure 3:**
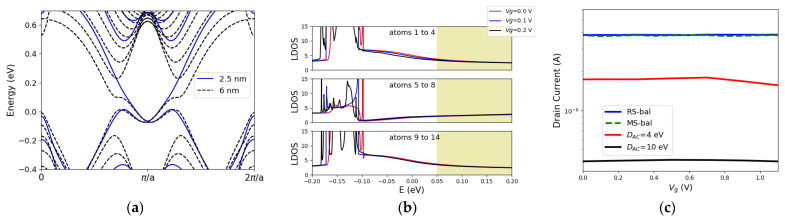
(**a**) Electronic band dispersion for 2.5 nm and 6 nm wide zigzag stanene nanoribbons. (**b**) LDOS in a gate-controlled 2.5 nm wide zigzag stanene nanoribbon. The contribution of atoms at the left edge (atoms 1 to 4), atoms at the center (atoms 5 to 8), and atoms at the right edge (atoms 9 to 14) are shown. (**c**) transfer characteristics for the ballistic (mode space vs. real space) and dissipative cases with *D*_AC_ of 4 eV and 10 eV.

Three major characteristics were linked to ultra-narrow Xene nanoribbons by using a Kane-Mele Hamiltonian [15]. First, a decrease in the width results in an increase in the bulk gap. Our DFT simulations are shown in Figure 3a exhibit an increase in the bulk gap from 0.21 eV for *W* = 6 nm to 0.52 eV for a *W* = 2.5 nm in a zigzag stanene ribbon. Second, the crossing point (Dirac point of the edge states) and the anti-crossing point (the momentum of the massive Dirac dispersion) are located at different momentum spaces. The non-trivial to trivial phase transition occurs as the crossing point moves towards the anti-crossing point and the gap opens once these points coexist at the same momentum space with a large overlap of states. The states in the momentum space in the vicinity of the anti-crossing point have finite inter-edge overlap at the same momentum space, which opens a gap. As confinement effects are introduced, the anti-crossing point moves closer to the crossing point at time-reversal invariant momenta (TRIM). This leads to a reduction in the critical electric field for topological phase transition.

Third, the gate-induced phase transition can occur without a bulk gap closing, owing to quantum confinement effects. Furthermore, in the ultra-narrow case, a gap appears before the edge states reach the conduction valleys. These three properties make ultra-narrow ribbons potential candidates for applications in topological devices.

It was shown in the previous section that the small bulk gap of stanene makes it unprotected against scattering that broadens the bulk states. It raises the question that whether an increase in the bulk gap of stanene (as an example of Xenes) with finite size effects can solve this problem. To answer this question, we explore the transport properties of ultra-narrow stanene ribbons.

We use a 2.5 nm wide zigzag ribbon of stanene as the channel material for the structure sketched in Figure 2a. The LDOS for varying gate voltages is illustrated in Figure 3b. The contribution of middle atoms and edge atoms are shown separately. It can be observed that, even though the bulk gap has increased, the states with higher energy in the bulk gap are not localized and the middle atoms are contributing to these states (the states in the shaded region in Figure 3b, covering from E = 0.05 eV to E = 0.2 eV). Therefore, even though the bulk state broadening will not cover the entire bulk gap, the poorly localized states allow scattering. Only a small energy window of 0.15 eV is well-localized at the edges, and the width of this window seems to be almost unaffected by the width variation of the ribbon. This results in a current degradation with scattering comparable to that of the 4 nm wide ribbon (Figure 3c), despite the fact that the delocalized states are at different momentum spaces (Figure 3a). This suggests that in ultra-narrow TIs, despite a larger bulk gap, the transport properties are not enhanced accordingly. However, the gate control could still be optimized with thinner ribbons due to the reduction of the critical field and the transition without the need for a bulk gap closing. Additionally, a simulation with a real-space Hamiltonian was performed to further confirm on current level the validity of our mode space simulations. As shown in Figure 3c, a good agreement was observed.

**Figure 4:**
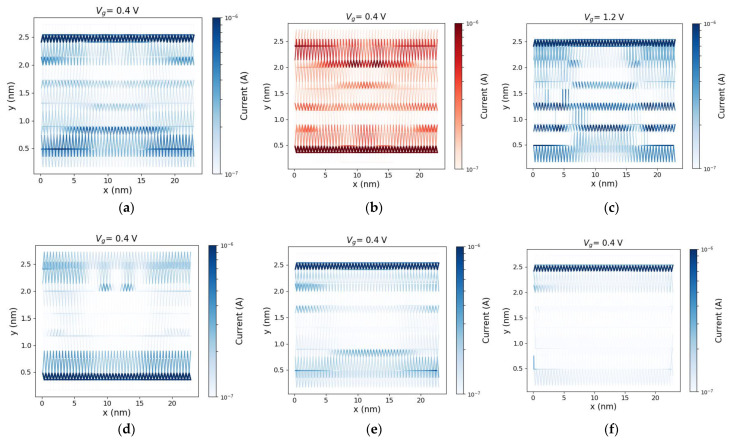
(**a**) The atom resolved current of forward moving spin-up electrons in the device under *V*_g_ = 0.4 V and *V*_DD_ = 0.05 V (the ballistic case), (**b**) same as (**a**) but for the spin-down electrons. (**c**): Same as (**a**) for *V*_g_ = 1.2 V. (**d**): Same as (**a**) but for backward moving spin-up electrons. (**e**,**f**): Same as (**a**) for *D*_AC_ = 4 eV and *D*_AC_ = 10 eV, respectively.

To study the effects of e-ph scattering on the helical edge-state transport, the spin-resolved atom-to-atom current is plotted in Figure 4. It is worth noting that *V*_DD_ = 0.05 V in this setup. It is shown that the forward moving spin up/down currents are mostly localized at the opposite edges (Figure 4a,b). However, there is a smaller portion of charge transfer taking place at the center of the ribbon as well as at the opposite edge. This indicates the contribution of the bulk and the opposite edge atoms to the spin-up/down states in the bulk gap. Figure 4c demonstrates that this contribution increases with *V*_g_ and the transport happens through more delocalized states. Figure 4d shows the backward moving spin-up carriers located at the opposite edge of that of the spin-up forward moving channel. The spin-momentum locking can be deduced from these results. As e-ph scattering is included (Figure 4e,f), the transfer of forward moving electrons is reduced at the edge and mostly in the middle of the ribbon, indicating the degradation of the current.

**Figure 5:**
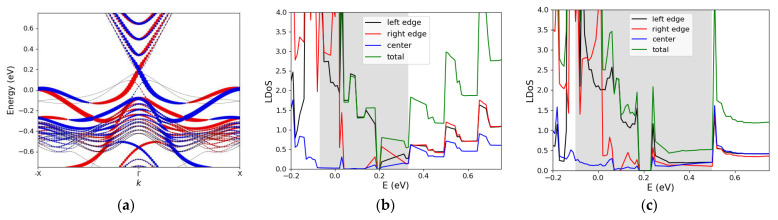
(**a**) Spin-resolved band structure of a 6.8 nm wide zigzag Na_3_Bi ribbon, projected on the left edge atoms. The red and blue dots indicate spin up and spin down states, respectively. (**b**) The contribution of the left/right edge atoms as well as the middle atoms to the LDOS of a 6.8 nm wide zigzag Na3Bi ribbon. (**c**) Same as (**b**) but for a 3.9 nm wide ribbon. The grey-shaded region illustrates the bulk gap.

In addition to stanene, monolayers and bilayers of Na_3_Bi have attracted much attention. Experiments have shown that Na_3_Bi monolayers have a bulk band gap of 360 meV, as well as a relatively small critical field of 1.1 V/nm [20]. TI-FETs based on ultra-short, ultra-narrow Na3Bi ribbons have been theoretically explored [16]. In a TI-FET relying on the gap opening as a switching mechanism, in addition to a small critical field, the band gap opened after the transition should be sufficiently large to efficiently obstruct the current. Previous works based on DFT have shown that for wider ribbons of Na_3_Bi (above 6 nm), the band gap opened after the transition is small (smaller than 0.2 eV). However, thinner Na_3_Bi ribbons have larger gaps (around 0.4 eV), post-topological phase transition. Therefore, it may be beneficial to use thinner Na_3_Bi ribbons to exploit the larger post-transition gap. Our results show that at a width of under 4 nm, the confinement effects result in a gap opening due to the overlap of states around the Fermi level. Additionally, although the bulk gap increases with a reduction in width (Figure 5b,c), the states in the bulk gap are poorly localized, akin to stanene (the grey-shaded region in Figure 5c). The opened gap and the larger contribution of the middle atoms to the states inside the bulk gap pose challenges to the use of ultra-narrow Na_3_Bi in a TI-FET. However, if a wider Na_3_Bi ribbon is used, the gap in the off state (after transition) is only around 0.2 eV, which seems challenging to have a low-leakage TI-FET operating based on electric-field-induced phase transition with Na_3_Bi.

### 3.3. Electric Field-Induced Topological Phase Transition in Xenes

Topological switching with gates is one of the mechanisms proposed for controlling transport in TIs. The topological switching can happen with a perpendicular out-of-plane electric field which closes and reopens the bulk gap. Another way to manipulate the edge state transport is through the application of an in-plane transverse electric field. Here, we study the effects of an applied lateral field on the transport and electronic properties of stanene and bismuthene nanoribbons.

**Figure 6:**
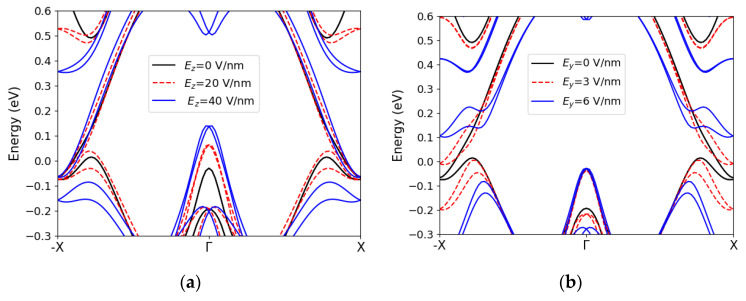

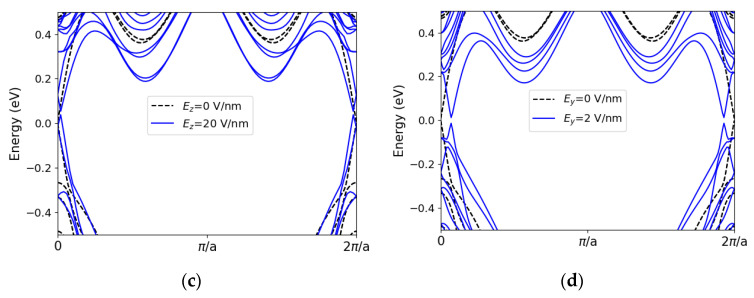
Band structure of a 2.5 nm wide stanene zigzag ribbon under (**a**) an out-of-plane electric field (**b**) a transverse electric field. Band structure of a 4 nm wide zigzag bismuthene ribbon under (**c**) an out-of-plane electric field (**d**) a transverse electric field.

Figure 6 illustrates the electric-field-induced changes in the band dispersion of stanene and bismuthene. Figure 6a shows that, by applying an out-of-plane field (*E_z_*), a gap opens at the X point in the edge states of the stanene zigzag ribbon However, the unprotected states around the Γ point move up into the gap as well. In Figure 6b, we see that the lateral field (*E*_y_) opens a gap at the X point as well, using an about 10× reduced field. The gap is around 0.13 eV for *E_y_* = 6 eV/nm. However, the states around Γ remain unchanged, as they are mainly formed by the middle atoms. In bismuthene, both lateral and out-of-plane fields shift the protected and unprotected states and close the bulk gap (Figure 6c,d). Once the bulk gap is closed, the spin-polarized states cannot be isolated anymore. Therefore, the transport will be unprotected. In addition, the lateral field seems to have a more drastic influence on the energy dispersion compared to *Ez* in bismuthene. It can be inferred that the lateral field can tune the band structure in a different way than the out-of-plane field, and in some materials, it can be more advantageous to use the lateral field.

**Figure 7:**
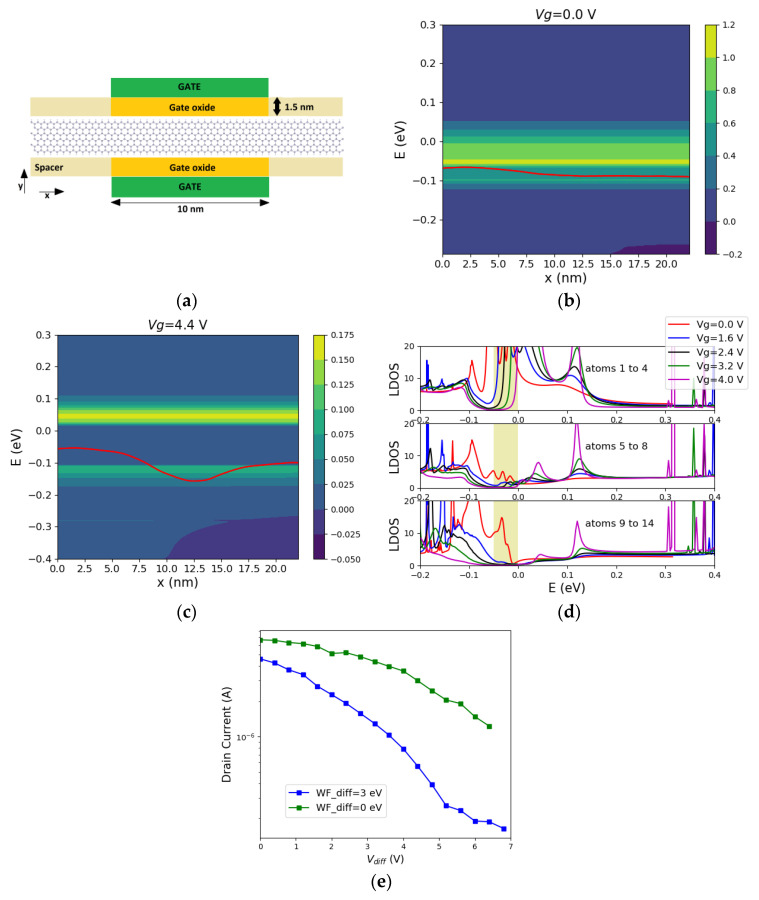
(**a**) Illustration of the structure with side gates imposing an electric field to a 2.5 nm wide zigzag stanene nanoribbon. *L*_ch_ = 10 nm and the gate oxide thickness is 1.5 nm. Current spectrum J(x,E) for the structure with side gates and a 2.5 nm wide zigzag stanene nanoribbon with (**b**) *V*_left_ = 0 (**c**) *V*_left_ = 4.4 V. In both cases *V*_right_ = 0 V, *V*_DD_ = 0.05 V. The red lines indicate the charge neutrality points in the middle of each slab. (**d**) The contribution of the left edge atoms (atoms 1 to 4), middle atoms (atoms 5 to 8) and the right edge atoms (atom 9 to 14) to the LDOS of the same structure. Here, the side gates are assumed to be made of metals with different work functions (work function difference (WF_dif = 3 eV). The yellow-shaded area shows the Fermi window. (**e**) IV curve of the structure with side gates with the gate metals having the same work function (WF_diff = 0) or different work functions (WF_diff = 3 eV).

A lateral field can be applied by having side gates, which we investigate next using zigzag stanene ribbons (Figure 7a). Here, stanene is chosen since the application of a lateral field opens a gap, which should be detectable in the LDoS obtained from our NEGF simulations.

Figure 7b,c show the energy-resolved current spectrum, for *V*_left_ = 0 and *V*_left_ = 4.4 V, respectively. The voltage on the other gate is *V*_right_ = 0 V. Additionally, *L_ch_* = 10 nm, *V*_DD_ = 0.05 V and a doping concentration of 3 × 10^20^ cm^−3^ is introduced to shift the Fermi level. A gap of around 0.1 eV is observed in the device with *V*_left_ = 4.4 V (Figure 7c), which confirms the opening of a gap by the external lateral electric field.

The LDOS in the central slab of the device is illustrated in Figure 7d. It is shown that as the electric field goes up, the edge channel states remain on one edge (see Figure 7d, top figure) and a gap opens in the LDOS (in the Fermi window highlighted by a shaded area in Figure 7d). Figure 7d shows that there is a finite value for LDOS in the Fermi window prior to the gap opening, indicating the presence of the metallic edge states in the Fermi window. As the electric field goes up, the LDOS disappears in the Fermi window (see Figure 7d at *V*_g_ = 4 V). As a result of this gap, the current decreases and an *I*_ON_/*I*_OFF_ ratio of around 28 can be observed at room temperature (Figure 7e).

By employing gate metals with different work functions, a built-in electric field is induced. This should reduce the threshold field required to open a gap. Figure 7d shows that at *V*_g_ = 0 V there is already a strong asymmetry in the LDoS formed by the left edge atoms compared to that of the right edge atoms in the Fermi window. This difference is due to the induced electric field from the side metal gates with different work functions. Figure 7e demonstrates that having a built-in electric field effectively decreases the threshold voltage needed for a gap opening. Hence, it can be a useful approach in ultra-thin TI ribbons (TIs with large bulk gaps), as the phase transitions happen without the bulk gap closing. However, it raises the question that whether the transport properties are favorable in a TI with a built-in electric field. In other words, are the spin-momentum locking and the protected transport preserved in a specific TI with a built-in field? This question requires more investigation.

## 4. Discussion

We studied the effects of electron–phonon coupling on the transport properties of two Xenes with non-trivial topological phases and different bulk band gaps. It was observed that in a stanene ribbon (a bulk gap of 0.17 eV), e-ph scattering caused considerable current degradation. This was because of the extension of the bulk states with a high density of states into the bulk gap. As a result, the edge localization of the protected states is destroyed, even with a small deformation potential. On the other hand, if we study the transport through the protected states of bismuthene (bulk gap of 0.5 eV), a significantly higher immunity to e-ph scattering is observed.

In previous work, it was proposed that an ultra-thin Xene ribbon benefits from an increase in the bulk gap, a reduction in the critical electric field, and a phase transition without a bulk gap closing [15]. An enlarged bulk gap can improve the edge state transport in stanene. Hence, we studied the quantum confinement effects on the transport properties of stanene zigzag ribbons. It was observed that, despite a significant increase in the bulk gap (from 0.21 eV for *W* = 6 nm to 0.52 eV for a *W* = 2.5 nm), only a small energy window of the states in the gap is adequately localized. Most states in the bulk gap are delocalized but at different momentum spaces. Similar conclusions were also drawn for Na_3_Bi. Once, e-ph scattering is included, electrons can backscatter. This results in almost no improvement in the immunity to electron–phonon scattering.

Finally, we studied the electronic and transport properties of TI ribbons subjected to lateral electric fields. It was shown that the lateral field can alter the band structure drastically, especially since the edge atoms form the protected metallic states. The application of *E*_y_ opened a gap in the metallic states of the stanene ribbon and closed the bulk gap in bismuthene. Utilizing this gap modulation, a stanene TI-FET with a channel length of 10 nm exhibits an *I*_ON_/*I*_OFF_ ratio of 28 at room temperature. The threshold voltage required to open a gap shows an improvement to the previously reported TI-FETs. The use of a built-in electric field can further reduce the threshold voltage. However, the effects of a built-in field on the transport properties of a TI warrants deeper investigation.

## Data Availability

The data presented in this study are contained within the article and are available on reasonable request from the corresponding author.
